# Cardiac magnetic resonance differences in ventricular function and delayed enhancement according to clinical presentation in Chagas disease

**DOI:** 10.1186/1532-429X-17-S1-P270

**Published:** 2015-02-03

**Authors:** Luis E Rodriguez Castellanos, Gabriela Melendez, Aloha Meave, Jorge I Magaña, Leyli C Velasquez Alvarez

**Affiliations:** Magnetic Resonance, National Institute of Cardiology, Mexico City, Mexico

## Background

Chagas disease is a parasitosis caused by the protozoan *Trypanosoma cruzi*. It is endemic in Latin America, nevertheless it has spread to other regions of the world due to migration. Up to 30% of the infected patients progress to the chronic phase, with a 5-year mortality of 50% due to heart failure or ventricular arrhythmias. We aimed to compare ventricular function and delayed enhancement (DE) according to the predominant clinical presentation in Chronic Chagas disease (CCD) either ventricular tachycardia (VT) or heart failure (HF), as well as to compare CCD with the undetermined and subclinical phases.

## Methods

Fifty-one patients with serologic diagnosis of Chagas disease underwent cardiac magnetic resonance (CMR) from 2009 to 2014 at our institution. Patients were classified as undetermined, subclinical and CCD, according to clinical, EKG and echocardiographic findings. CCD patients were further subdivided according to the predominant clinical presentation into those with VT or HF.

## Results

Out of 51 patients, 6 were classified as undetermined, 5 subclinical and 40 with CCD (21 VT and 19 HF). Myocardial scar expressed as mass and percentage of myocardium was similar between patients with VT and those with HF (44.5 ± 20.1 g. vs. 48.6 ± 30.5 g. p=0.62 and 43.8 ± 13% vs. 46.4 ± 19.9%, p=0.64 respectively). There was significant difference in DE distribution, basal lateral wall (both anterolateral and inferolateral) was affected in 91% of patients with VT compared to 55% of those with HF, p=0.02. Patients with VT had higher left ventricular ejection fraction than patients with HF (36.3 ± 10.9% vs. 23.5 ± 10.6%, p <0.001) and lower end-systolic volume [99 ml (IQR 75-158) vs 153 ml, (82-193); p = 0.02]. Compared with HF patients those with VT had higher right ventricular ejection fraction [43% (31-49) vs 26% (18-45); p = 0.03] and lower right end-diastolic and end-systolic volumes [68 ml (58-96) vs 100 ml (71-171), p = 0.01 and 36 ml (32-67) vs 68 ml I (42-129), p = 0.004 respectively].

## Conclusions

There was no significant difference in DE burden between patients with VT or HF in CCD; however the DE distribution was significantly different, with the lateral wall more frequently involved in VT group. HF patients had biventricular lower ejection fraction with higher end-systolic and end-diastolic volumes.

## Funding

None.

**Figure 1:**
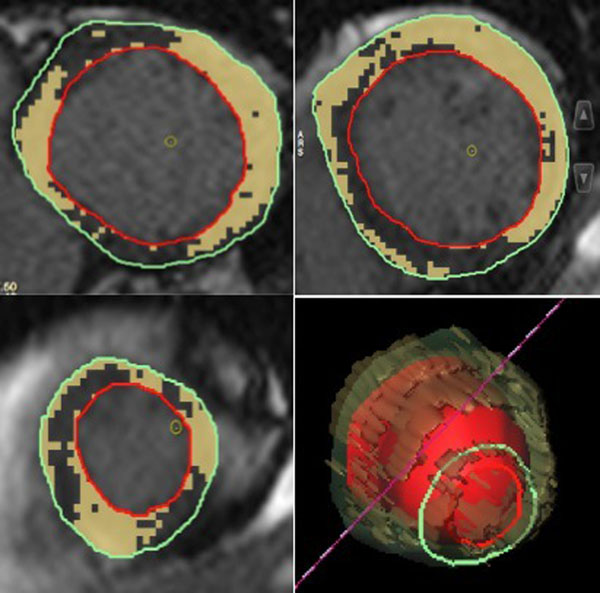
Figure 1

**Table 1:** Differences according predominant to clinical presentation in Chronic Chagas Disease.

	With VT (n=21)	Witout VT (n= 19)	p
Male sex, n(%)	14 (66)	8 (42)	0.20
Age (years)	58.5 ± 12.7	55.6 ± 13.4	0.48
EDLVD (mm)	57 (54-76)	63 (57-69)	0.08
ESLVD (mm)	46 (40-61)	54 (48-64)	0.14
LVEF(%)	36.3 ±10.9	23.5 ± 10.6	<0.001
LVEDV (ml )	165 (148-221)	191 (132-230)	0.16
LVESV (ml)	99 (75-158)	153 (82-193)	0.02
Left Ventricular Mass (g)	88 (85-114)	106 (87-144)	0.13
RVEF (%)	43 (31-49)	26 (18-45)	0.03
RVEDV (ml)	68 (58-96)	100 (71-171)	0.01
RVESV (ml)	36 (32-67)	68 (42-129)	0.004
Ventricular Aneurism n(%)	6 (28)	3 (16)	0.46
Thrombus	1 (5)	6 (32)	0.04
Left Anterior Fascicular Block n(%)	1 (4)	3 (16)	0.33
Segments with delayed enhacement	8.9 ±3.0	9.3 ±3.6	0.69
Fibrosis grade (Cuantitative Delayed Enhacement) (gr)	44.5 ±20.2	48.6 ± 30.5	0.62
Percentage of fibrosis (delayed enhacement %)	43.8 ±13.0	46.4 ± 19.9	0.64

